# The worrying, current state of addictions training in medicine

**DOI:** 10.3389/fmed.2022.1062096

**Published:** 2022-11-24

**Authors:** Robert M. Lundin, Harry Hill

**Affiliations:** ^1^Institute for Mental and Physical Health and Clinical Translation (IMPACT), Deakin University, Geelong, VIC, Australia; ^2^Change to Improve Mental Health (CHIME), MHDAS, Barwon Health, Geelong, VIC, Australia; ^3^Department of Mental Health, Albury Wodonga Health, Wangaratta, VIC, Australia

**Keywords:** addictions, drugs, alcohol, training, dual diagnosis, competency, curriculum

## Introduction

Over 10 years ago, a systematic review by Rasyidi et al. (1) highlighted grave concerns about the split between addiction medicine and addiction psychiatry in the USA. It also expressed concerns about gaps in the curriculum and competencies from undergraduate education through to residency. The review argued that the integration of addiction training should be primarily focused outside of psychiatry and made a strong argument for standardizing the assessment of the training provided using validated scores to measure progress (1). Another review by Ayu et al. (2) demonstrated that the literature only holds descriptions of the curriculum and training structure for a handful of countries (United States, Canada, Australia, United Kingdom, Germany and Netherlands), identifying significant gaps in our knowledge of how addictions are taught (2).

Since then, substance use and addiction have remained unaddressed problems in medicine. Previous large-scale public health policies, such as three decades of “war on drugs” in the USA, have drained public resources and failed to impact the prevalence of addictions significantly (3). The World Drug Report 2021 demonstrates that the potency of substances is increasing while the public perception of risk from use is decreasing. This development follows a global increase in substance use by 22% over the past decade. There are also grave concerns around unexpected global events such as a four-fold increase in access to illicit substances obtained from “the dark web” during the COVID-19 pandemic (4).

Attempts to estimate the global financial cost of drug abuse poses many challenges and often fail the capture the wider issues. However, the catastrophic impacts on individuals are well documented and include physical and mental health conditions, ranging from infectious and respiratory disease to trauma and depression. Most concerning, considering the lack of international policy developments to tackle it, is that drug use is currently the sixth most common cause of death in 15–49-year-olds (5). While there is an increasing awareness of mental health problems, it has not yet become common to address the similar stigma associated with addictions in public and medical settings.

## We can't treat what we can't see

International research consistently demonstrates the high prevalence of drug and alcohol morbidity among patients presenting for health care services. A large audit of patients presenting to Australian emergency departments found that over one-third of patients had a drug and alcohol problem that was contributing to their current presentation and requiring intervention. Unfortunately, these concerns are frequently unidentified, increasing the risk of inappropriate treatment and patient management. This also makes it difficult for hospital services to plan sufficient interventions (6).

Whilst there is a clear need for specialist care regarding addictions, it is vital to remember that drug and alcohol issues are not bespoke, and all healthcare professionals should have essential core competencies in addiction. Unfortunately, the exposure to addictions in medical school is variable, often not entering curriculums until after graduation. Ayu et al. have proposed a set of basic addiction core competencies for doctors, highlighting the appropriate knowledge, attitudes, and skills in addiction that doctors should achieve at each education level. While specific areas of knowledge are not outlined in detail, these skills are vital to the provision of holistic, evidence-based healthcare (7).

A focus on theory alone will not be sufficient. Lubman et al. demonstrated that despite acknowledgment of the need for varied health professionals to undertake training and “expand the curriculum”, the limited clinical exposure and appropriate supervision regarding working with people living with primary and co-occurring substance use problems amplifies the evident issues around recognition, screening and ultimately the ability to provide effective intervention (8).

There are some recent examples of improving postgraduate exposure to addiction. The 2021 Royal Commission into Victoria's Mental Health System helped introduce mandatory psychiatry rotations for all junior medical staff and has been hailed as a positive development in increasing mental health knowledge and reducing stigma (9). While an important step, there is a clear need for a similar development for addictions. As such, early work in integrating mental health and addiction services could be introduced at the medical student level. It has been demonstrated that spending one of six weeks of a psychiatry rotation focused on addiction will increase knowledge of substance use. This should be considered an acceptable trade-off considering the study also showed that the overall gains from the mental health component were not lost (10).

## Hearing the stories

A persistent theme in addiction is the lack of health professional knowledge. It is well established that this often reinforces stigma and a lower prioritization of addiction as a core issue in medicine. In the UK, nursing students completing a 3-year program demonstrated no increase in knowledge and skills of addiction between the beginning and end of their degree. While this likely represents a lack of structured training and education, it was further found that the clinical experiences they had with patients had negatively affected the “therapeutic commitment.” This is an even greater concern because stigma appears to be increasing when clinical contact lacks relevant supervision, reflection and education. It remains unclear if the attitudes seen are generated from the clinical experience directly or passed on from senior staff members, which would be helpful information in targeting intervention (11).

Importantly, the current zeitgeist of addiction is shifting. The Rethink Addiction campaign in Australia is an independent campaign representing a collaborative industry effort to reinvent addiction services through evidence-based information and linkages to support. Rethink Addiction was formed to educate and advocate for the need to change Australia's attitude and approach to addiction. Key to addressing the widespread and damaging misunderstandings of addiction and the associated stigma is the encouragement of those with lived and living experiences to share their stories of hope and recovery. Sharing the real stories of addiction demonstrates that this complex issue does not only touch all humans but reinforces that help is available and change is possible.

It is postulated that exposing health professionals to these stories can function as a primer to eliciting a cultural change in medicine. This becomes increasingly important considering the effects of clinical exposure without context as outlined above. A starting point would be to better see addiction as a core competency in medicine and something that all health professionals have a duty to provide evidenced based interventions for.

## Specialist pathways

There is currently no established single pathway for training an addiction physician. In the United States, addiction medicine and addiction psychiatry have been listed as medical specialities accredited by the Accreditation Council for Graduate Medical Education since 2015 and 1993 with 1- and 2-year training fellowships, respectively (12, 13). In other countries, like the United Kingdom and Australia, addiction training is primarily split between psychiatry, physician and primary care (family medicine) training colleges with varying degrees of collaboration and standardization of curriculums (14). These training colleges face important debates regarding whether addiction training should be more generalized to all psychiatrists or a specialist concern. Despite this, addiction problems and their consequences are equally seen and managed by pain specialists (particularly opioids), emergency physicians (overdoses and intoxication) and general medicine (detoxification and complications from use). This means that upskilling the current workforce needs input from all these medical stakeholders (12).

While most members of the public will look to the psychiatrist as an addiction expert, most training programs only have limited coverage of addictions. In a survey of psychiatric trainees in 30 European countries, only 59% had training in drugs and alcohol. Of these, 43% reported problems with their training. This is a particular concern due to the significant variations in training opportunities, systems and curricula across Europe, demonstrating a greater role for regional organizations (15). The situation is even worse in low- and middle-income countries, which are simultaneously seeing the most rapid growth in the prevalence of drug use and the availability of addiction specialists will be even less. The solution can, therefore, not rely on doctors alone (4, 16). Similar gaps are seen in general internal medicine residents in the USA, where the majority report being unprepared to treat substance use disorders (17).

## Large and rapid changes

Outdated curriculums and limited exposure to substance use disorders do not only make the medical workforce unfit for the current landscape. On the horizon loom a wide range of new developments, challenges and treatments that require understanding and involvement in legislative developments and policies. Most recently, the treatment option of long-acting injectable buprenorphine (LAIB) is moving through countries as a game-changer in the management of opioid use disorder. It remains unclear how LAIB affects other co-occurring addictions and prescribing is still often limited to specialist treatment providers as opposed to primary care. Overall, LAIB provides a wide range of benefits from reduced cost, stigma, improved stability across multiple domains and allows more time to engage in society (18).

Following increasingly widespread use in medicine and psychiatry, Virtual Reality interventions have just started exploring treatment options for addictions, such as cue exposure therapy for substance cravings. While promising, virtual therapies have yet to fully identify all potential adverse effects, which needs increased focus. Similarly, functional neuroimaging is beginning to uncover how addictive behaviors alter brain structure and functioning (19). This development parallels the only recently recognized internet gaming disorder, which is a behavioral addiction with minimal treatment options worldwide. More concerningly, this is increasingly diagnosed even before new gaming technologies with enhanced immersion, like mixed realities, have reached full maturity (20).

On the horizon is the use of psychedelic drugs through microdosing alone or in combination with psychotherapy, where several studies are currently providing the relevant evidence base to consider efficacy (21). The next step for psychedelic treatments will be the consideration of legislative changes if clinical use is to occur. If this process is left to politicians and people with vested interests alone, there is a substantial risk of laws and policies being made without sufficient evidence underlining them. It is, therefore, essential that research is carried out by independent academic institutions that can facilitate measured, impartial discussion in weighing up benefits and harm to counter the reduced perceived harm by the general public (4).

Mental health and neuroscience are increasingly utilizing the concept of Brain Capital as a common framework that can explain the consequences of inaction on matters that affect the brain health of the wider population. This concept helps reduce stigma, explain the impact of disorders, target investment and capacity building from businesses and organizations and promotes an evidence-based approach to public policy development. Using such frameworks can potentially help the general public understand that addiction is not only a problem for the individual patient but rather a societal issue to be addressed. Addiction medicine would benefit greatly from the lessons learned from Brain Capital in framing how illicit substance use and addictions impact the cognitive reserve, workforce availability and overall well-being of the population. It could also help communicate the financial consequences of not acting now as a driver for addressing the rapid changes outlined above (22). An overview of the challenges faced is outlined in Table 1.

**Table 1:** Overview of key challenges facing addiction training in medicine.

**Challenges in addictions training**
No agreed training pathway	Addiction training is currently split between primary care and psychiatry, (12, 13) with limited collaboration on core competencies in addiction (7, 14)
Workforce education	There are identified gaps in addiction training for nurses, (11) medical students (10) and general medicine (17) and psychiatry trainees (15) surrounding knowledge of disorders and the competency and confidence in treating them
Substance use disorders not identified	Outdated training, often due to rapid changes, means that substance use problems are not identified in clinical settings (6, 8)
Novel therapeutics	Development of new interventions such as virtual reality (19) and long-acting injective buprenorphine (18) means the treatment landscape is rapidly changing and requires a continuing medical education focus
New diagnoses on the horizon	New technology means the introduction of new addictive behavioral disorders like Internet gaming disorder (20)
Psychedelics	Re-ignited focus on the use of psychedelics in psychiatry means legislative and clinical challenges will need more research around microdosing, use with psychotherapy with need for a measured debate (21)
Common framework that defies stigma	Old anti-drug campaigns have failed and there is need for new frameworks, such as brain capital, to describe addictions with a focus on stories and lived experiences (22)

## Discussion

In this article, we have outlined how the two fields of addiction medicine and addiction psychiatry lack not only an internationally recognized structure but also that substance use disorders are not satisfactorily taught in undergraduate and postgraduate medicine and nursing. While over 10 years have passed since the last systematic review of addiction training, the issues identified remain unaddressed, while the prevalence of substance use has worsened with new challenges and an outdated curriculum. We urgently need a full review of international developments since then. It will also be equally important to develop a greater understanding of the structure of addiction training outside the Western countries mentioned in order to observe international trends and guide development.

Coordinated work between medical specialities, training organizations and universities is needed to establish a training framework to implement already identified core competencies while allowing for the incorporation of rapidly changing developments like new treatments, technologies and legislation. Though some core competencies have already been suggested, further work should detail the specific knowledge needed. New technology, like machine learning, might help identify and flag substance use problems in key clinical settings to guide intervention and outline key learning targets.

Purposeful and well-coordinated educational and clinical opportunities, combined with thoughtful exposure to stories of recovery, will be powerful tools to lead to sustained change and evidence-based care. The concept of Brain Capital provides one possible framework for shifting public opinion on addictions. These changes need to translate into clear, evidence-based public health policies that address the stigma associated with substance use disorders and addictions.

## Author contributions

HH conceptualized article. RL wrote first draft and. Both authors edited and approved final manuscript.

## Conflict of interest

The authors declare that the research was conducted in the absence of any commercial or financial relationships that could be construed as a potential conflict of interest.

## Publisher's note

All claims expressed in this article are solely those of the authors and do not necessarily represent those of their affiliated organizations, or those of the publisher, the editors and the reviewers. Any product that may be evaluated in this article, or claim that may be made by its manufacturer, is not guaranteed or endorsed by the publisher.

## References

[1] Rasyidi E Wilkins JN Danovitch I. Training the next generation of providers in addiction medicine. Psychiatr Clin North Am. (2012) 35:461–80. 10.1016/j.psc.2012.04.001 22640766

[2] Ayu AP Schellekens AFA Iskandar S Pinxten L De Jong CAJ. Effectiveness and organization of addiction medicine training across the globe. Eur Addict Res. (2015) 21:223–39. 10.1159/000381671 25966903

[3] King RS Mauer M. The war on marijuana: the transformation of the war on drugs in the 1990s. Harm Reduct J. (2006) 3:6. 10.1186/1477-7517-3-6 16469094 PMC1420279

[4] United Nations. World Drug Report 2021. New York City, NY: United Nations publication (2021).

[5] International Narcotics Control Board,. Economic Consequences of Drug Abuse (2013). Available online at: https://www.incb.org/documents/Publications/AnnualReports/Thematic_chapters/English/AR_2013_E_Chapter_I.pdf (accessed September 15, 2022).

[6] Butler K Reeve R Viney R Burns L. Estimating prevalence of drug and alcohol presentations to hospital emergency departments in NSW, Australia: impact of hospital consultation liaison services. Public Health Res Pract. (2016) 26:2641642. 10.17061/phrp2641642 27714385

[7] Ayu AP El-Guebaly N Schellekens A De Jong C Welle-Strand G Small W. Core addiction medicine competencies for doctors: an international consultation on training. Subst Abuse. (2017) 38:483–7. 10.1080/08897077.2017.1355868 28718723 PMC5788448

[8] Lubman DI Hides L Jorm AF Morgan AJ. Health professionals' recognition of co-occurring alcohol and depressive disorders in youth: a survey of Australian general practitioners, psychiatrists, psychologists and mental health nurses using case vignettes. Aust N Z J Psychiatry. (2007) 41:830–5. 10.1080/00048670701579090 17828656

[9] State of Victoria. Royal Commission into Victoria's Mental Health System, Final Report, Summary and Recommendations, Parl Paper No. 202, Session 2018–21. (2021). p. 120.

[10] Christison GW Haviland MG. Requiring a one-week addiction treatment experience in a six-week psychiatry clerkship: effects on attitudes toward substance-abusing patients. Teach Learn Med. (2003) 15:93–7. 10.1207/S15328015TLM1502_04 12708066

[11] Clancy C Oyefeso A. Getting addiction into the nursing education “water supply”: A U. K case study. J Addict Nurs. (2019) 30:149–58. 10.1097/JAN.0000000000000295 31478962

[12] Haque LY Fiellin DA. Bridging the gap: dual fellowship training in addiction medicine and digestive diseases. Dig Dis Sci. (2022) 67:2721–6. 10.1007/s10620-022-07478-9 35430700 PMC9013212

[13] Nunes EV Kunz K Galanter M O'Connor PG. Addiction psychiatry and addiction medicine: the evolution of addiction physician specialists. Am J Addict. (2020) 29:390–400. 10.1111/ajad.13068 32902056

[14] Klimas J. Training in addiction medicine should be standardised and scaled up. BMJ. (2015) 351:h4027. 10.1136/bmj.h4027 26220548 PMC4707523

[15] Orsolini L Rojnić Palavra I Papanti GD Potočan M Quattrone D Martens M. Psychiatry trainees' attitudes, knowledge, and training in addiction psychiatry-A European survey. Front Psychiatry. (2021) 11:585607. 10.3389/fpsyt.2020.585607 33488419 PMC7820719

[16] Balhara YPS Sharma P Chawla N. Education and training on addiction psychiatry in low and middle income countries: Observations from existing literature and recommendations going ahead. Asia-Pac Psychiatry. (2021) 13:e12492. 10.1111/appy.12492 34873858

[17] Wakeman SE Baggett MV Pham-Kanter G Campbell EG. Internal medicine residents' training in substance use disorders: a survey of the quality of instruction and residents' self-perceived preparedness to diagnose and treat addiction. Subst Abuse. (2013) 34:363–70. 10.1080/08897077.2013.797540 24159907

[18] Shulman M Wai JM Nunes EV. Buprenorphine treatment for opioid use disorder: an overview. CNS Drugs. (2019) 33:567–80. 10.1007/s40263-019-00637-z 31062259 PMC6585403

[19] Segawa T Baudry T Bourla A Blanc J-V Peretti C-S Mouchabac S. Virtual Reality (VR) in assessment and treatment of addictive disorders: a systematic review. Front Neurosci. (2020) 13:1409. 10.3389/fnins.2019.01409 31998066 PMC6965009

[20] Zajac K Ginley MK Chang R. Treatments of internet gaming disorder: a systematic review of the evidence. Exp Rev Neurotherap. (2020) 20:85–93. 10.1080/14737175.2020.1671824 31544539 PMC6930980

[21] Berkovitch L Roméo B Karila L Gaillard R Benyamina A. Efficacy of psychedelics in psychiatry, a systematic review of the literature. L'Encephale. (2021) 47:376–87. 10.1016/j.encep.2020.12.002 33888297

[22] Eyre HA Ayadi R Ellsworth W Aragam G Smith E Dawson WD. Building brain capital. Neuron. (2021) 109:1430–2. 10.1016/j.neuron.2021.04.007 33957073 PMC8240507

